# Co-Encapsulation of Coffee and Coffee By-Product Extracts with Probiotic *Kluyveromyces lactis*

**DOI:** 10.3390/foods13193056

**Published:** 2024-09-26

**Authors:** Dérica Gonçalves Tavares, Mayara Andrade Martins de Souza, Tamara Leite dos Santos, Adriele do Amor Divino Silva, Danilo José Machado de Abreu, Whasley Ferreira Duarte

**Affiliations:** 1 Department of Biology, University of Louisville, Louisville, KY 40292, USA; dericatavares@gmail.com; 2 Department of Biology, Federal University of Lavras, Lavras 37200-000, MG, Brazil; mmandradesouza@gmail.com (M.A.M.d.S.); tamaraleitesantos@gmail.com (T.L.d.S.); adriele2704@gmail.com (A.d.A.D.S.); danilo.mabreu@gmail.com (D.J.M.d.A.)

**Keywords:** bioactives, alginate, microcapsules, yeasts

## Abstract

Coffee and coffee by-products contain several chemical compounds of great relevance, such as chlorogenic acid (CGA), trigonelline, and caffeine. Furthermore, yeasts have been the target of studies for their use as probiotics because of their interesting biochemical characteristics. The combined administration of probiotic microorganisms with components that provide health benefits mediated by alginate encapsulation is an alternative that ensures the stability of cells and chemical compounds. In this context, the aim of this work was to co-encapsulate the probiotic yeast *Kluyveromyces lactis* B10 and extracts of green coffee beans, coffee silverskin, and PVA (black, green or immature, and sour coffee beans). The bioactive composition, antioxidant and antimicrobial activities of the extracts, microcapsule morphological characteristics and encapsulation efficiency, ability of the encapsulation to protect the yeast cells subjected to gastrointestinal conditions, and antioxidant activity of the microcapsules were evaluated. All the evaluated extracts showed antioxidant activity, of which PVA showed 75.7% and 77.0%, green coffee bean showed 66.4% and 45.7%, and coffee silverskin showed 67.7% and 37.4% inhibition of DPPH and ABTS^•+^ radicals, respectively, and antimicrobial activity against the pathogenic bacteria *E*. *coli*, *Salmonella*, and *S*. *aureus*, with high activity for the PVA extract. The microcapsules presented diameters of between 1451.46 and 1581.12 μm. The encapsulation efficiencies referring to the yeast retention in the microcapsules were 98.05%, 96.51%, and 96.32% for green coffee bean, coffee silverskin, and PVA, respectively. Scanning electron microscopy (SEM) showed that the microcapsules of the three extracts presented small deformations and irregularities on the surface. The *K. lactis* cells encapsulated in all treatments with the extracts showed viability higher than 8.59 log CFU/mL, as recommended for probiotic food products. The addition of green coffee bean, coffee silverskin, and PVA extracts did not reduce the encapsulation efficiency of the alginate microcapsules, enabling a safe interaction between the extracts and the *K. lactis* cells.

## 1. Introduction

Coffee is one of the most consumed beverages in the world and Brazil is the largest producer of coffee beans, with production expected for 2023/24 of 66.3 million bags of green bean equivalent [1]. Depending on the post-harvest process, several coffee by-products are generated, such as green coffee beans, coffee pulp, parchment, coffee silverskin, mucilage, and spent coffee grounds [2,3]. Green coffee beans include all naked coffee beans before roasting [4], with a water content in dry coffee seeds of up to 10–12% [5]. Green coffee beans are then roasted to produce coffee. Green coffee beans contain compounds with biological activity, among them caffeine and CGA, which are the most studied due to their effects on the human body [6].

The occurrence of defective coffee grains resulting from problems during harvesting and pre-processing can affect around 15 to 20% of the total coffee produced in Brazil, and their presence together with other beans is capable of reducing the final quality of the beverage [6,7]. In Brazil, these defective coffee grains are commonly called PVA, which came from the Portuguese “preto” (black beans), “verde” (green or immature beans), and “ardido” (sour beans), and they are the main defects found in coffee beans [8]. PVA like commercial green coffee beans contain several chemical compounds and CGA, caffeine, and trigonelline are the most relevant constituents.

Coffee silverskin is another by-product generated during the roasting process and is characterized as a film that covers the coffee bean [9]. Coffee silverskin represents around 4.2% of the total weight of the seed and one ton is generated for every 120 tons of processed coffee [10]. Coffee silverskin has been proposed as a new functional ingredient due to its prebiotic and antioxidant capacities [11]. According to [12], coffee silverskin contains flavonoids, CGA, and caffeine in its composition, comprising more than 30 bioactive compounds.

Coffee and some of its by-products contain bioactive compounds that are currently recognized as being of great interest in research and industry. The compound that is most widely used is chlorogenic acid, whose health benefits include controlling blood pressure and cholesterol, regulating blood sugar levels, and providing antioxidant action against free radicals.

The extraction of bioactive compounds is one of the alternatives for recovering active compounds, such as CGA, caffeine, and trigonelline, present in coffee by-products. Such compounds have shown potent physiologically stimulating effects, such as antioxidant, anti-inflammatory [13], antidiabetic [14], and anti-obesity [15] activities, and research also suggests the antimicrobial [16] and immunomodulatory [17] potential of these molecules. These metabolites, which have important biological activities for human health, such as antioxidant and antimicrobial activities, could be used in the production of functional foods.

In addition to the importance of functional food production, interest in probiotics has also grown in recent years. Probiotics are defined as food supplements composed of live microorganisms that confer benefits to the health of the host when consumed in adequate quantities [18]. *Kluyveromyces lactis* and *Torulaspora delbrueckii,* isolated from Canastra cheese by our research group, demonstrated viability after exposure to simulated gastrointestinal tract (GIT), as well as promising results regarding self-aggregation, hydrophobicity, β-galactose activity, and antibiotic resistance [19,20]. *K. lactis* and *T. delbrueckii* are also considered safe according to hemolysis and mucin degradation tests, as well as showing positive results for the co-aggregation of pathogens, and *K. lactis* showed 90% animal survival rate against salmonellosis in in vivo testing using mice [21].

Microencapsulation is an interesting tool in providing a protected environment for microorganisms and bioactive compounds, as it releases them in a controlled manner, ensuring stability [22]. This process has been shown to improve the viability of probiotic bacteria during passage through the GIT [23], and maintenance of the bioactive compounds CGA and caffeine obtained from coffee silverskin [24]. Here, we co-encapsulated the probiotic yeast *K. lactis* B10 with extracts of green coffee bean, coffee silverskin, and PVA and evaluated the microcapsule morphological characteristics, encapsulation efficiency, ability of the encapsulation to protect yeast cells subjected to gastrointestinal conditions, and the maintenance of the extract antioxidant activity. We also analyzed the bioactive composition and the antioxidant and antimicrobial activities of the extracts.

## 2. Materials and Methods

### 2.1. Extraction of Bioactive Compounds from Green Coffee Beans, Coffee Silverskin, and PVA Beans

The coffee and by-products used were provided by Cooxupé–Cooperativa Regional de Cafeicultores em Guaxupé, harvested and processed in the 2022 harvest in the Southern Region of the State of Minas Gerais/Brazil. The coffee beans, PVA, and silverskin were stored in a cold chamber at 10 °C before use. The extraction of bioactive compounds was carried out according to Suárez-Quiroz et al. [25], with some modifications. Individual solutions of green coffee bean, PVA, and coffee silverskin were prepared. Each raw material was ground separately in an IKA A11 analytical mill. Finally, the grain size of the materials was standardized using 200 mesh sieves. Each sample containing 100 g of substrate was mixed with 500 mL of 70% aqueous ethanol and stirred in a shaker for 24 h at room temperature in the absence of light. The solutions were vacuum filtered in a Whatman nº1 qualitative filter and the ethanol was evaporated in a rotary evaporator at 60 °C at 120 rpm (IKA—RV 10). The aqueous extracts obtained were dried in an oven at 60 °C (the temperature used for CGA isolation [25]) for 2 days and analyzed by high-performance liquid chromatography (HPLC).

### 2.2. High-Performance Liquid Chromatography Analysis of the Bioactive Compounds

The concentrations of CGA, caffeine, and trigonelline in the extracts were determined by HPLC according to Malta and Chagas [26] and Santiago et al. [27]. The analyses were carried out using a Shimadzu Liquid Chromatograph (Shimadzu Corp., Kyoto, Japan) equipped with a quaternary pump model LC-20 AT, degasser model DGU-20 A5, automatic injector model SIL-20A HT, column oven model CTO-20 A, UV detector model SPD-20A, and interface module model CBM-20A. The chromatographic column used was the Agilent-Zorbax Eclipse XDB-C18 (4.6 × 250 mm, 5 μm) connected to an Agilent-Zorbax Eclipse. Elution was carried out using an isocratic mode consisting of an 85% solution of 1% glacial acetic acid in type 1 water (Milli-Q) and 15% methanol. The elution flow was 1 mL/min, the injection volume was 20 µL, and the detector was operated at 272 nm. The chromatograms obtained were analyzed and treated with LC Solution software 5.3 (Shimadzu). The identification of compounds was carried out by comparing their retention times with the retention times of certified standards, and quantification was carried out using external calibration curves [28]. All samples were analyzed in duplicate.

### 2.3. Inoculum Preparation and Encapsulation of Yeast and Extracts

The yeast *K. lactis* B10 isolated from Canastra cheese by Andrade et al. [19] was reactivated in 1 mL of YPD (1% yeast extract, 2% peptone, and 2% glucose) and incubated at 30 °C for 24 h. The culture was centrifuged at 7000 rpm for 10 min and the cell concentration was adjusted to 109 cells/mL. Encapsulation was performed according to Sathyabama et al. [29], with some modifications. The mixture was prepared by combining 0.5 g sodium alginate, 2 g extract (green bean, coffee silverskin, or PVA bean), 2 mL *K. lactis* B10 at 10^9^ CFU/mL, and 0.02 mL Tween 80 in a final volume of 100 mL. The mixture was stirred vigorously for 30 min until complete emulsion. Using a 5 mL syringe, the mixture was dropped into a 0.1 mol/L calcium chloride solution from a distance of 10 cm, remaining in this solution for 30 min. The spheres were separated by aseptic filtration, washed with 0.009 g/mL saline solution containing 5% glycerol and stored at 4 °C. The diameter of the freshly prepared microcapsules was measured using the Nikon SMZ 1500 Fluorescence Stereoscopic Microscope from the Electronic Microscopy and Ultrastructural Analysis Laboratory—LME, UFLA.

#### 2.3.1. Encapsulation Efficiency

The encapsulation efficiency of *K. lactis* B10 was determined according to Ladislau et al. [30], with some modifications. One gram of microcapsules, after production (T0), was dissolved in 9 mL of phosphate buffer (0.1 mol/mL, pH 7.0), followed by homogenization for 10 min. An aliquot of 100 μL from the dissolution of microcapsules was spread on a YPD plate and CFU/mL was determined after 48 h of incubation at 30 °C.

The efficiency value of encapsulated yeast was evaluated using the equation

(1) $$EE\%=\frac{N}{N_{0}}\times 100$$

where *N* represents the number of yeast cells released from the microcapsules, and *N*_0_ the initial inoculum number used in the encapsulation process.

#### 2.3.2. Scanning Electron Microscopy (SEM)

For SEM, the microcapsules were fixed in Karnovsky solution (2.5% glutaraldehyde, 2.0% paraformaldehyde in 0.05 M sodium cacodylate buffer, 0.001 M CaCl_2_, and pH 7.2) and stored at 4 °C for 24 h. After fixation, the samples were washed 3 times for 10 min each in 0.05 M cacodylate buffer, followed by dehydration in different acetone gradients (25%, 50%, 75%, 90%, and 100%), remaining for 10 min at each concentration and 3 times for 10 min at 100% concentration. The samples were dried in a Balzers CPD 030 critical point dryer, mounted on metal supports (Stubs), and metallized with gold in a Balzers SCD 050 sputter coater. The samples were observed using a TESCAN CLARA field-emission scanning electron microscope (Zeiss) from the Electronic Microscopy and Ultrastructural Analysis Laboratory—LME, UFLA.

#### 2.3.3. Confocal Laser Microscopy

Confocal laser microscopy of the microcapsules was performed to observe the presence of *K. lactis* B10 and to assess its vitality by the live–dead test. Therefore, 5 μL of depolymerized microcapsules in sterile water were added to a water agar block and the live–dead test was performed according to Moreira et al. [31]. Propidium iodide is a DNA-binding dye that penetrates the cell membranes of dead or dying cells and has been used to stain dead cells. Syto9 dye stains both living and dead cells and was used to stain living cells. The imagens were acquired using the LSM780 Zeiss Observer Z.1 Confocal Laser Microscope (Carl Zeiss, Jena, Germany) with Zen 2010 software from the Electronic Microscopy and Ultrastructural Analysis Laboratory—LME, UFLA. For the excitation of propidium iodide, an HeNe 543 ηm laser line with an emission filter range of 625–665 ηm was used, and for the excitation of Syto9, an Argon 488 ηm laser line with an emission filter range of 520–550 ηm was used.

### 2.4. Probiotic Yeast Survival under Simulated Gastrointestinal Conditions

The microcapsules, after 10 days of storage at 4 °C, were subjected to simulated gastrointestinal (GI) tract conditions containing synthetic gastric and duodenal juices, according to Andrade et al. [20]. One gram of microcapsules was added to 10 mL of gastric juice (6.2 g/L NaCl, 2.2 g/L KCl, 0.22 g/L CaCl_2_, 1.2 g/L NaHCO_3_, 0.3% pepsin, and pH 3.0) and incubated at 37 °C with shaking. After 90 min, 17.5 mL of synthetic duodenal juice (6.4 g/L NaHCO_3_, 0.239 g/L KCl, 1.28 g/L NaCl, 0.1% pancreatin, 10% ox bile, and pH adjusted to 7.4 with 5 M HCl) were added and stirred at 150 rpm for 180 min at 37 °C, simulating passage through the intestinal tract. The yeast survival rate was evaluated at 0 (T0), 90 (T1), and 270 (T2) minutes by plating 100 μL in YPD medium and incubating at 37 °C for 48 h [20].

### 2.5. Determination of Antibacterial Activity

The antibacterial activity of the extracts was determined according to Gudiña et al. [32]. The pathogenic bacteria used were *S. aureus* ATCC 25923, *S. enterica* ATCC 5190, and *E. coli* EPEC ATCC 055. The minimum inhibitory concentration (MIC) was determined by microdilution in a 96-well polystyrene microplate. The pathogenic bacteria strains were reactivated in BHI broth (brain and heart infusion broth) and incubated at 37 °C for 24 h. Bacterial suspensions were prepared in saline solution (0.85% NaCl) according to McFarland’s nephelometric scale 1, which comprises 3 × 108 colony-forming units per mL (CFU/mL). The concentrations of extracts tested in the experiment were 50, 25, 12.5, and 6.25%. Aliquots of 10 μL of the bacterial inoculum were added to the wells containing 140 μL of the BHI solution and extract (final inoculum concentration of 2 × 107 CFU/mL), totaling 150 μL per well. The microplates were incubated at 37 °C for 24 h. In this experiment, wells without coffee extract were used as negative controls and chloramphenicol at 1 mg/mL was added as a positive control. For each type of extract and concentrations tested, a blank was inserted, allowing intrinsic absorbance to be discounted according to the extract colors.

The experiment was carried out in triplicate for the concentrations of each extract and for each bacterial isolate. Absorbance readings were performed on a microplate reader (BioTek Powerwave XS, Winooski, VT, United States) at 600 nm. The percentages of growth inhibition in different concentrations of coffee extracts for each microorganism were calculated from the following equation:

(2) $$\%Growth\;inhibition=[1-\left(\frac{A_{c}}{A_{0}}\right)]\times 100$$

where A_c_ is the absorbance of the coffee extract in different concentrations and A_0_ is the absorbance of the control without coffee extract. The MIC of each extract was determined as the lowest extract concentration that completely inhibits measurable bacterial growth (A_600_ = 0).

### 2.6. Determination of Antioxidant Activity

The antioxidant activity of the extracts of green coffee beans, coffee silverskin, and PVA beans, and of the depolymerized microcapsules, was evaluated by the DPPH (1,1-diphenyl-2-picrylhydrazyl) and ABTS^•+^ (2′-azino-bis-3-ethylbenzthiazoline-6-sulphonic acid) radical scavenging methods.

The extracts in concentrations of 5, 2.5, 1.25, 0.62, and 0.31 mg/mL were diluted in distilled water. The microcapsules at concentrations of 100, 50, 25, 12.5, and 6.25 mg/mL were depolymerized in 0.1 mol/L phosphate buffer and centrifuged at 6000 rpm for 20 min and the supernatant was used in the tests [33].

The DPPH radical scavenging method was performed according to Tavares et al. [34] and the ABTS^•+^ radical scavenging method according to Tavares et al. [34]. The negative and positive controls used were ethyl alcohol and 0.05% Trolox, respectively. The experiment was carried out in triplicate. The percentage of inhibition (I%) of the DPPH and ABTS^•+^ free radicals was calculated using the equation below:

(3) $$I\%=[\frac{A_{blank-}A_{sample}}{A_{blank}}]\times 100$$

where A_blank_ is the absorbance of the negative control and A_sample_ is the absorbance of the analyzed extracts. The results were also subjected to 50% effective concentration (EC_50_), expressed as the minimum concentration of antioxidant necessary to reduce the initial concentration of DPPH and ABTS^•+^ radicals by 50%. A curve with the values of I% plotted on the *y*-axis and the concentration of the extracts (mg/mL) on the *x*-axis was obtained, and the curve of the line was determined by the equation below:

(4) y=ax+b

where *y* is the percentage inhibition of the DPPH and ABTS^•+^ radicals, and *x* is the EC_50_ (mg/mL).

### 2.7. Determination of Total Phenolic Compounds

The total phenolic compounds of the extracts of green coffee beans, coffee silverskin, and PVA beans and of the depolymerized microcapsules were determined by the Folin–Ciocalteu method, according to Tavares et al. [34]. The extract diluted in distilled water and the depolymerized microcapsules in 0.1 mol/L phosphate buffer at a concentration of 1.5 mg/mL were used. The total phenolic compounds were calculated from the straight-line Equation (5) of the gallic acid standard curve, and the results were expressed as mg of gallic acid equivalent (GAE) per g of extract. The experiment was carried out in triplicate.

(5) $$\begin{array}{c} y=0.0315x-0.0223\\ R^{2}=0.9921 \end{array}$$

### 2.8. Statistical Analysis

The results were subjected to analysis of variance (ANOVA) using the R Statistics program, and the means were compared using the Scott–Knott test at *p* < 0.05 level of significance [35].

## 3. Results and Discussion

### 3.1. Analysis of the Bioactive Compounds from Green Coffee Beans, Coffee Silverskin, and PVA Beans

Green coffee bean, coffee silverskin, and PVA bean extracts were evaluated for their contents of CGA, caffeine, and trigonelline (Table 1). A high CGA concentration was found in PVA bean extract with 223.02 mg/g that was statistically different (*p* < 0.05) from green coffee bean extract with 204.72 mg/g. However, only 10.83 mg/g of CGA was observed in coffee silverskin extract. PVA also comes from coffee beans, which explains the high CGA and caffeine contents, which are close to the values observed for only green coffee beans.

The higher CGA content in defective beans (PVA) compared to green coffee beans reported in this work has also previously been described in the literature [8,36], while the CGA concentration extracted from green coffee beans was higher than has been reported in the literature [37,38,39]. A high CGA content in defective beans was found by Habtamu and Belay [36] using aqueous extract, and by Kalchne et al. [8] in steam-treated PVA. These extracts come from a mixture of black, green, and sour beans, which is why the high CGA and caffeine contents are close to those observed for green coffee beans. PVA contains coffee beans in different physiological stages and the CGA content in coffee decreases as the fruit ripens, showing different concentrations of CGA between mature and fully ripe fruits [40], which may be related to the high CGA content in PVA. CGA, caffeine, and trigonelline concentrations are also directly affected by the extraction method used, which may explain the different concentrations found in other studies. Therefore, the extraction method used in our work was shown to be efficient in CGA extraction from PVA and green coffee beans. PVA can represent up to 20% of coffee production and its selection and separation from healthy beans is necessary to maintain the marketing quality of superior coffees. However, PVA has a lower price compared to green coffee beans and was the byproduct that presented more bioactive compounds, which shows the importance of using PVA in biotechnological preparations, such as microcapsules.

Coffee silverskin extract showed a low CGA concentration of 10.83 mg/g (Table 1) that was similar to the value found by Bessada et al. [41] of 11.7 mg/g. CGA is a water-soluble compound with low thermal stability, making it easily degraded during the roasting process, which may explain the low concentration found in our work since coffee silverskin is obtained from the roasting process of green coffee beans. However, considering the amount of this by-product generated in the coffee industry and the fact that the industry discards this by-product, we must emphasize that this is still a source of CGA to be considered.

Trigonelline concentration was higher and statistically different in coffee silverskin extract (4.38 mg/g) compared to green coffee bean (1.04 mg/g) and PVA bean (0.79 mg/g) extracts (Table 1). Trigonelline, similarly to CGA, has low thermal stability and during roasting process it is easily degraded in aroma and flavor [42]. Wang et al. [43] found relative high contents of trigonelline in unroasted (9.7%) and roasted (5.6%) coffee silverskin. Trigonelline has also been shown to have several biological activities, such as hypoglycemic, neuroprotective, anti-invasive, estrogenic, and antibacterial activities [44]. Anti-aging activity has also been reported and trigonelline may delay the development of neurodegenerative diseases in *C. elegans* [45] and recover memory function in Alzheimer’s disease in model mice [46]. Although trigonelline is one of the most abundant compounds found in coffee beans, low concentrations of trigonelline were observed for green coffee bean and PVA extracts. The possible reasons for this may be related to the extraction method. Enzymatic extraction methods have demonstrated superior results for trigonelline concentrations in green coffee bean extracts [47].

The caffeine content was higher (*p* < 0.05) in PVA bean extract with a 145.51 mg/g concentration and statistically different from green coffee bean and coffee silverskin extracts with 116.59 mg/g and 62.04 mg/g concentrations, respectively (Table 1). A caffeine concentration of 62.04 mg/g was the most significant result from three bioactive compounds studied for silverskin. This fact can be explained because caffeine is the only one among the bioactives that is not easily degraded during roasting. Even though coffee silverskin is a by-product that is subjected to the roasting process, the result demonstrates that there was good extraction of this compound by the method used. High caffeine compositions in defective beans compared to non-defective beans were also described by Prandi et al. [48]. Generally, high caffeine content is linked to coffee beans quality [49]. However, in our work, a higher caffeine content was observed in PVA than in green coffee beans, which may be due to the presence of coffee beans in different physiological states compared to green coffee beans only. There are studies showing changes in the concentrations of CGA, trigonelline, and caffeine in coffee beans throughout the development of cherries [50,51]. Although, PVA is not suitable for composing higher quality coffees, it is a by-product rich in phenolic compounds as it is a mixture of damaged and green coffee beans [48]. Caffeine in coffee silverskin extract was observed in high concentrations compared to CGA and trigonelline, probably because caffeine does not degrade easily during the coffee roasting process [52]. Our results for caffeine concentration in coffee silverskin extract were higher than those reported by Bessada et al. [41]. This difference may be related to the extraction methods, geographic differences in cultivation, and the types of coffee used.

### 3.2. Determination of Antibacterial Activity

The use of agroindustry by-products as a natural source of bioactive compounds responsible for antimicrobial and antioxidant activity represents an environmentally and economically attractive option, given consumer concerns about the replacement of these synthetic, active compounds in industry [53]. Considering the current importance of using alternative sources of bioactives with antimicrobial activity, the activity of green coffee bean, silverskin, and PVA extracts were determined by measuring the percentage of inhibition of the growth of pathogenic bacteria.

Green coffee bean, coffee silverskin, and PVA extracts at 50% (500 mg/mL) concentrations were able to inhibit all bacterial growth (Table 2). The PVA extract showed high antibacterial activity, where the MBC and MIC for *S. aureus* were 6.25% (62.5 mg/mL) of extract and for *Salmonella* and *E. coli* it was 25% (250 mg/mL) of extract. The high antimicrobial activity of PVA extract may be due to higher concentrations of CGA and caffeine (Table 1). Studies suggest that the effect of the antimicrobial and antioxidant activities of the hydroxyl groups of CGA act especially by disturbing the permeability of bacterial cell membranes [54].

The PVA extract also had a high concentration of caffeine and it is known that caffeine acts by inhibiting protein synthesis and causing damage to the DNA of bacteria [55]. According to Duangjai et al. [56] the antimicrobial activity of coffee pulp extract was attributed to CGA, caffeine, quinic acid, and malic acid. Considering the MBC, the PVA extract showed more promising antibacterial activity compared to the other extracts. This characteristic may be due to this by-product not being subjected to the roasting process, therefore maintaining the initial concentration of compounds in its composition, which are different from those reported for green coffee bean extract.

The MIC and MBC of the green coffee bean extract for *Salmonella* and *E. coli* were 50% of the extract and for *S. aureus* they were 25% of the extract (Table 2). CGA and caffeine have been reported in the literature to exhibit antimicrobial activity [12] and are present in high concentrations in green coffee bean extract. However, the minimum concentration of green coffee bean extract with bactericidal activity was 250 mg/mL, which may be high when compared to other studies. The work of Tasew et al. [57] and Lin et al. [58] showed that 31.25 mg/mL and 4.69 mg/mL of green coffee bean extract, respectively, were able to inhibit the growth of *S. aureus*. Although caffeine, trigonelline, and CGA are more abundant in coffee extracts [12], other compounds, like alkaloids and polyphenols, may be also responsible for antibacterial activity in green coffee bean extract.

The MIC and MBC of the coffee silverskin extract for *Salmonella* and *E. coli* were 25% of extract; however, for *S. aureus*, it showed good bacteriostatic effect with 6.25% of extract, although the MBC was 50% of extract. Coffee silverskin is the only by-product among the extracts studied that is subjected to the roasting process, and it is understood that these high bacteriostatic and bactericidal activity values are not due to the loss of compounds due to high roasting temperatures. In a study by Nzekoue et al. [12], none of the coffee silverskin extracts expressed activity only with an extract concentration greater than 512 mg/L, making its use as a crude extract unfeasible, which highlights the effectiveness of the extraction method used in this work.

All three extracts affected the Gram-positive bacteria *S. aureus* more than the Gram-negative bacteria *E. coli* and *Salmonella*. The difference in the action of the extracts between Gram-positive and Gram-negative bacteria may be due to the structures and external walls of Gram-negative bacteria. Low-molecular-weight phenolic compounds diffuse into the cytoplasm of bacterial cells, causing acidification and subsequent cell death. The mechanism of the antibacterial activities of compounds with action on the cytoplasmic membrane and inhibition of intracellular and extracellular enzymes is inhibited in Gram-negative bacteria such as *E. coli* and *Salmonella* due to the presence and conformation of their outer membranes [55,58].

### 3.3. Antioxidant Activity and Total Phenolic Compounds of Extracts

Considering the relevance of polyphenols and antioxidants commonly present in coffee and its by-products, the antioxidant activity of green coffee bean, coffee silverskin, and PVA extracts was evaluated using the DPPH and ABTS methods, which provide a comprehensive analysis of the total antioxidant capacity, covering different types of radicals. Furthermore, a quantitative test of total phenolic compounds was also carried out for green coffee bean, coffee silverskin, and PVA extracts using the Folin–Ciocalteu method.

**Figure 1:**
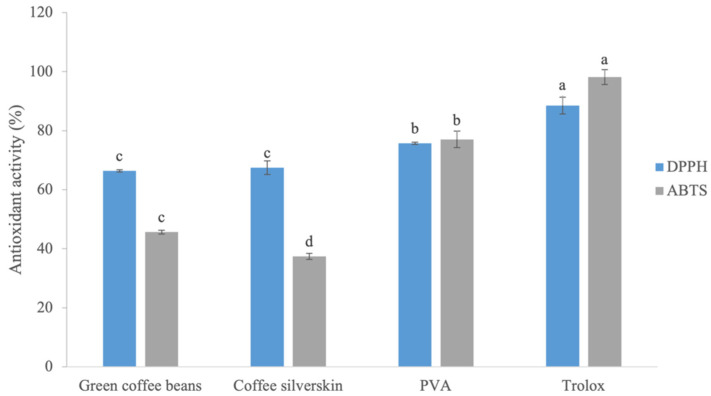
Antioxidant activity of green coffee bean, coffee silverskin, and PVA extracts by the 1-diphenyl-2-picrylhydrazyl (DPPH) and 2′-azino-bis-3-ethylbenzthiazoline-6-sulfonic acid (ABTS^•+^) methods. Data are expressed as triplicate mean ± standard deviation. Means with different letters are significantly different at *p* < 0.05 by Scott–Knott test.

The PVA extract showed higher antioxidant activity and was statistically different from the other extracts, with 75.7% and 77.0% inhibition of DPPH and ABTS^•+^ radicals, respectively (Figure 1). The inhibition of DPPH radicals by green coffee bean and coffee silverskin extracts was statistically lower than that found for PVA, but they still present a significant percentage of inhibition of DPPH radicals of 66.4% and 67.7%, respectively. The inhibition of ABTS^•+^ radicals by the green coffee bean and coffee silverskin extracts was also lower than for the PVA extract, but they still showed a considerable inhibition percentage of 45.7% and 37.4%, respectively (Figure 1).

The PVA extract presented the highest concentration of total phenolic compounds of 78.1 mg of GAE/g of extract, which is in accordance with its high antioxidant activity (Table 3). However, these percentages of free radical inhibition do not always reflect the amounts of phenolic compounds, considering that not all phenolic compounds have radical scavenging activity. We can observe this when we examine the green coffee bean extract, which showed greater antioxidant activity but lower phenolic concentration (19.4 mg of GAE/g of extract) when compared to the coffee silverskin extract (23.4 mg of GAE/g of extract). Silva et al. [59] evaluated the antioxidant potential and total phenolic concentration of coffee by-product extracts and observed values of 84.95% and 92.81% inhibition of DPPH and ABTS^•+^ radicals, respectively, and 97.89 mg of GAE/g for phenolic compounds. These values are higher than those reported in our work, probably due to the different extraction methods used, although the values were similar for the PVA extract. Other studies have shown the presence of phenolic compounds in plant by-products, such as avocado seeds and peels [60], pecan shells [61] and mango by-products [62], highlighting their potential use and low costs.

Another common method of expressing the efficiency of free radical inhibition by extracts is the EC_50_ method, which assesses the concentration of antioxidant substances in a solution capable of reducing by 50% the oxidative power of substances such as DPPH and ABTS^•+^ radicals (Table 3). PVA extract showed EC_50_ values of 0.6 and 3.2 mg/mL for DPPH and ABTS^•+^, respectively; statistically higher than the other extracts and indicating the strong free radical scavenging capacity of this extract. Coffee silverskin extract exhibited EC_50_ values for DPPH and ABTS^•+^ radicals of 3.6 and 6.8 mg/mL, respectively; also demonstrating that very low concentrations of these extracts are already sufficient to exert inhibitory activities in vitro (Table 3). This was also true for green coffee bean extract, where the value obtained was 5.9 mg/mL for ABTS^•+^.

### 3.4. Microcapsule Characterization of Encapsulation Efficiency

Considering the results obtained for the extracts regarding the presence of CGA, caffeine, and trigonelline and also the antioxidant and antimicrobial potential of these extracts, they were used in the composition of microcapsules in an alginate matrix together with the yeast *K. lactis* B10 previously characterized by our group as potentially probiotic [20,21]. Among the parameters evaluated in encapsulation, the diameter of the microcapsules is one of the most important characteristics because, depending on the components incorporated into the alginate and resulting in the matrix, this attribute can affect the solubility, survival, and release of cells during passage through the GI tract [63]. The average diameter of coffee silverskin microcapsules was 1581.12 µm, followed by green coffee bean with 1529.49 µm and PVA with 1451.46 µm (Table 4), showing a probable slight interference of the extracts used.

Differences in microcapsule sizes are common and have been reported in the literature [63,64,65]. The diameter of the microcapsules is an important characteristic because it depends on the components incorporated into the alginate and this attribute can affect solubility, survival, and cell release during passage through the GI tract [63]. A large reduction in microcapsule size can lead to loss of encapsulant protection, and an increase in size can cause a drop in the digestibility of pancreatic enzymes, impairing the release of probiotic cells [65]. Although small and medium sizes ranging from 0.2 to 1000 µm are most often reported, for encapsulation of probiotic cells as a delivery technology, large microcapsule sizes greater than 1000 µm have also been reported [63,64,65]. Furthermore, sizes greater than 1000 µm may also be associated with microcapsule preparation using the extrusion method, which depends on the diameter of the needle and the distance between the release of the alginate solution and the gelling solution [66].

**Figure 2:**
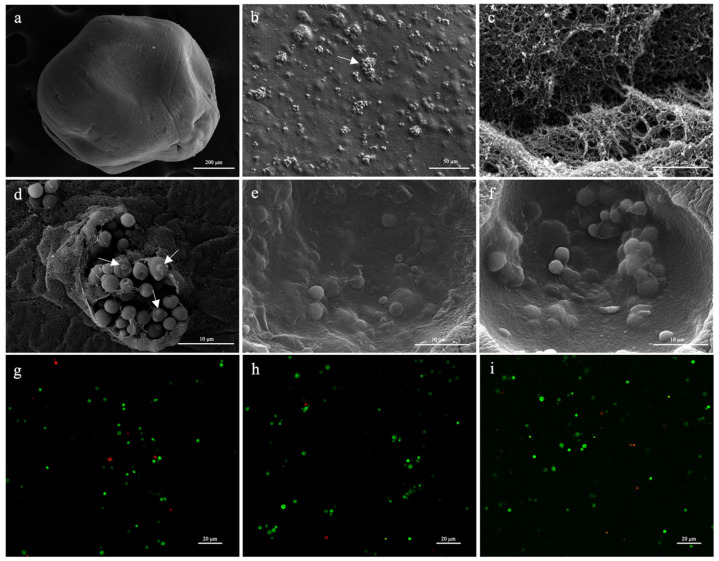
SEM and fluorescence microscopy of microcapsules. (**a**) External structure of the microcapsule; (**b**) distribution of *K. lactis* B10 in the microcapsule in groups of cells (arrow); (**c**) detail of the alginate porous network; (**d**) microcapsule with green coffee bean extract showing *K. lactis* B10 group of cells and cells that have undergone budding (arrows); (**e**) microcapsule with coffee silverskin extract showing *K. lactis* B10 group of cells; (**f**) microcapsule with PVA extract showing *K. lactis* B10 group of cells; (**g**–**i**) viability of *K. lactis* B10 encapsulated by the live–dead test. Dead cells were stained with Propidium Iodide (red) and living cells were stained with Syto9 (green). Microcapsules with green coffee bean extract (**g**), PVA extract (**h**), and coffee silverskin extract (**i**).

The morphological characteristics of the microcapsules were analyzed using SEM. The microcapsules from the three extracts presented similar morphology, as shown by the presence of small deformations and irregularities on the surface (Figure 2a), and it was possible to observe that the yeast was successfully encapsulated in alginate using the extrusion technique with green coffee bean (Figure 2d), coffee silverskin (Figure 2e), and PVA (Figure 2f) extracts. The irregularities observed in the microcapsules may be due to the SEM fixing method and the sample manipulation. Another cause of these irregularities may be the alginate concentration. Sakoui et al. [67] found that microcapsules with 3% alginate have a smooth surface with fewer pores, unlike microcapsules with concentrations close to 1% alginate, which have an irregular surface like those in this study.

In all microcapsules, the yeasts were distributed mostly grouped together (Figure 2b,d–f), which may be due the presence of the extracts, unlike in a study by Sathyabama et al. [29], in which bacteria were randomly distributed in the alginate matrix. Another interesting aspect observed was the porous structure of the alginate (Figure 2c), which is a very striking characteristic of this component and originates in the cross-linking arrangement between the alginate and Ca^2+^ during the process of gelation. Roquero et al. [68] suggest that the size of the pores in the structure depends on the concentration of the alginate used. The characteristics of irregularity and maintenance of cell viability were like those in the study carried out by Adilah et al. [69].

The encapsulation efficiency was evaluated and is shown in Table 4. No statistical difference was observed between the different extracts, and the high efficiency of 98.05–96.32% indicates good entrapment in the alginate matrix incorporated with the extracts and yeast cells. The relationship between viable cells before and after encapsulation is an important parameter for evaluating the interaction between the components incorporated into the microcapsule and the probiotic microorganism. Other studies have observed different results to ours, in that the composition of the microcapsule and the presence of extra protective agents affected the encapsulation efficiency (Raddatz et al., 2022; Bakhtiyari et al., 2022 [70,71]). In the study carried out by Petraitytė and Šipailienė [72], the extrusion technique showed the lowest encapsulation efficiency. However, in our study, microcapsules produced by the extrusion technique with calcium chloride as a crosslinking agent showed high efficiency with great retention of *K. lactis* B10 cells for subsequent release. Furthermore, the addition of green coffee bean, coffee silverskin, and PVA extracts did not reduce the encapsulation efficiency of the alginate microcapsules and formed safe interactions with *K. lactis* B10 cells.

### 3.5. Antioxidant Activity and Total Phenolic Compounds of Microcapsules

Phenolic compounds, responsible for exerting antioxidant activities such as those present in our extracts, tend to modify the sensory characteristics of the foods to which they are inserted, mainly creating bitterness and astringency. Within this context, microcapsules are a great alternative, as they preserve certain sensory characteristics and provide controlled delivery of such compounds [73]. The antioxidant activities of green coffee beans and PVA microcapsules were, respectively, 31.32% and 18.03% for DPPH radicals and 45.65% and 11.64% for ABTS^•+^ radicals. Regarding phenolic compounds, coffee silverskin, PVA, and green coffee beans showed 20.4, 15.9, and 19.6 mg of GAE/g of microcapsule, respectively. When analyzing the concentration of total phenolics and the antioxidant activity of the microcapsules, they showed lower values when compared to the crude extracts. However, we must consider that to evaluate the antioxidant activity and total phenolic compounds, the microcapsules were depolymerized and resuspended in a volume of 9 mL, which is equivalent to a 10-fold dilution of the extract. Even so, we can observe the percentages of antioxidant activity of the microcapsules containing PVA and green coffee bean extract.

The microcapsules containing the coffee silverskin extract did not present antioxidant activity, even though these microcapsules presented a considerable concentration of phenolic compounds (20.4 mg of GAE/g of microcapsule). In this study, analysis was carried out for DPPH and ABTS radicals, both reported in the literature as being efficient in identifying the activity of CGA and caffeic acid mainly, but both are methods of analyzing antioxidant activity based on electron transfer. Therefore, such results cannot be extrapolated to correspond to the total antioxidant activity of a sample. It is recommended to use more identification methods for other groups of compounds, based on hydrogen atom transfer for example [74,75]. Furthermore, the lower antioxidant activity of coffee silverskin microcapsules may be due to the interaction between the extract and the alginate used for encapsulation. The strong interaction or absorption between the phenolic compounds in the extract and the alginate may have hindered the release of these compounds, making interaction with free radicals difficult [76,77]. Tests with different concentrations of alginate and coffee silverskin extract would be necessary to better understand this interaction [76].

In recent years there have been studies incorporating these extracts into edible microcapsules such as those tested in the present work [78,79,80,81,82]. Sarabandi et al. [79], Romanini et al. [80], and Muhammad et al. [81] tested the incorporation of extracts from eggplant peel, grape pomace, and cinnamon bark into gum arabic and maltodextrin, alginate, and xanthan gum microcapsules. Radünz et al. [76] also tested microcapsules containing clove essential oil. When evaluating the antioxidant activity of microcapsules, some authors have observed a decrease in phenolic compounds and antioxidant activity, as occurred in our study.

Therefore, our results showed that PVA extract, green coffee bean extract and silverskin are promising in reducing the oxidative power of free radicals and have antibacterial activity. Furthermore, the addition of green coffee bean, silverskin, and PVA extracts did not reduce the encapsulation efficiency of the *K. lactis* B10 strain in alginate microcapsules, allowing delivery of the probiotic yeast plus antioxidant and antibacterial metabolites.

### 3.6. Survival in Gastrointestinal Conditions

The survival of *K. lactis* B10 co-encapsulated with green coffee bean, coffee silverskin, and PVA extracts was evaluated after microcapsule production (T0) and after passing through simulated gastrointestinal conditions (T1).

No statistical difference was observed in cell viability between T0 and T1 (after GI simulation). The population of *K. lactis* B10 went from 8.93 log CFU/mL (T0) to 8.76 log CFU/mL (T1) in green coffee bean extract microcapsules, 8.87 log CFU/mL to 8.75 log CFU/mL in coffee silverskin extract microcapsules, and 8.85 log CFU/mL to 8.59 log CFU/mL in PVA extract microcapsules.

The cell viability of *K. lactis* B10 after 10 days of storage at 4 °C (T0) was observed using SEM and fluorescence microscopy. Figure 2d displays yeast cells that were budding and the presence of scars from the release of daughter cells (arrows), which highlights their viability. Figure 2g–i display the viability of *K. lactis* B10 in the microcapsules with green coffee bean (Figure 2g), PVA (2 h), and coffee silverskin (Figure 2i) extracts by the live–dead test. The live–dead test shows a high concentration of live cells, stained with Syto9 (green), and low concentration of dead cells, stained with propidium iodide (red), for all treatments.

Studies on the co-encapsulation of green coffee bean, coffee silverskin, and PVA extracts with potentially probiotic yeasts are scarce in the literature. Our results demonstrate that the presence of extracts in the microcapsules did not interfere with the viability of yeast cells during microcapsule storage and after the simulation of passage through the GI tract. Andrade et al. [20] and Andrade et al. [21] also reported that the *K. lactis* B10 strain, shows high viability after passing through the GI tract. They found a cell survival rate of 81.5% and 1.42 × 10^8^ CFU/g in mouse feces in the in vivo test with *K. lactis* B10. Studies on co-encapsulation have shown high cell survival rates of the probiotic bacteria *Lactobacillus helveticus* [83], encapsulated in green tea, and *Lactobacillus rhamnosus* [84]. Encapsulation of the yeasts *Pichia barkeri* VIT-SJSN01, *Yarrowia lipolytica* VIT-ASN04, *Wickerhamomyces anomalus* VIT-ASN01, and *Saccharomyces cerevisiae* VIT-ASN03 with alginate also showed high survival cell rates by the extrusion method [85].

We observed that the alginate matrix gave the microcapsules mechanical resistance and good cell propagation in the pores, which resulted in high survival rates during storage and after simulated passage through the GI tract. According to Puscaselu et al. [86], alginate, in addition to providing mechanical resistance, is a cheap encapsulating agent, has chemical stability, and can reduce the perception of the unpleasant taste of the compounds inserted into its structure. Furthermore, the survival results observed in our work were higher than when an additional chitosan coating is used with yeast and probiotic bacteria [85,87]. Our results showed that there is no need for an extra coating for the encapsulation of probiotic yeast, making the microcapsules in this work a good low-cost alternative for supplying viable probiotic yeast using the extrusion technique, which is a simple and cheap method compared to others.

## 4. Conclusions

Our results showed that the addition of green coffee bean, coffee silverskin, and PVA extracts did not reduce the encapsulation efficiency of alginate microcapsules. Even with larger microcapsule diameters, safe interaction between the extracts and *K. lactis* B10 cells was possible. The encapsulation technique used in our work was shown to be a promising alternative as a delivery system for probiotic microorganisms and bioactive compounds. This technique promoted an adequate release of these components for possible fresh consumption or application in other food products, making them functional foods. As the results obtained in in vitro experiments cannot be extrapolated to the in vivo situation, evaluations of the resistance of the yeast *K. lactis* B10 to the GI tract of mammals, its persistence and dissemination, and the safety of its use as a probiotic, using animal models will be performed in the near future.

## Figures and Tables

**Table 1:** Concentrations of bioactive compounds in green coffee bean, coffee silverskin, and PVA bean extracts.

Extracts	Chlorogenic Acid (mg/g)	Caffeine (mg/g)	Trigonelline (mg/g)
PVA	223.02 ± 0.51 ^a2^	145.51 ± 3.47 ^a2^	0.79 ± 0.09 ^a1^
Green coffee bean	204.72 ± 3.64 ^a1^	116.59 ± 0.20 ^a3^	1.04 ± 0.08 ^a1^
Coffee silverskin	10.83 ± 0.06 ^a3^	62.04 ± 0.42 ^a1^	4.38 ± 0.01 ^a2^

Data are expressed as the means of duplicates. Means with different numbers are significantly different at *p* < 0.05 by Scott–Knott test.

**Table 2:** Minimum inhibitory concentrations (MICs) and minimum bactericidal concentrations (MBCs) of green coffee bean, PVA, and coffee silverskin extracts against food borne bacterial pathogens.

Extract	Green Coffee Bean			Coffee Silverskin			PVA		
	*Salmonella*	*S. aureus*	*E. coli*	*Salmonella*	*S. aureus*	*E. coli*	*Salmonella*	*S. aureus*	*E. coli*
50%	100 ± 0 ^aA^*	100 ± 0 ^aA^	100 ± 1 ^aA^*	100 ± 0 ^aA^	100 ± 0 ^aA^*	100 ± 0 ^aA^	100 ± 0 ^aA^	100 ± 0 ^aA^	100 ± 1 ^aA^
25%	49 ± 3 ^bC^	100 ± 0 ^aA^*	93 ± 3.5 ^bB^	99 ± 0 ^aA^*	100 ± 0 ^aA^	100 ± 2 ^aA^*	100 ± 0 ^aA^*	99 ± 0.6 ^aA^	98 ± 2 ^aA^*
12.5%	38 ± 0.4 ^cC^	41 ± 1 ^bB^	59 ± 0.2 ^cC^	87 ± 2.4 ^bA^	100 ± 0 ^aA^	99 ± 0.3 ^aA^	74 ± 0.7 ^bB^	98 ± 0.9 ^aA^	87 ± 0 ^bB^
6.25%	35 ± 1 ^cA^	28 ± 2 ^cC^	46 ± 1.1 ^dB^	20 ± 1.5 ^cC^	100 ± 0 ^aA^	24 ± 1 ^bC^	30 ± 0.4 ^cB^	97 ± 1 ^bA^*	66 ± 0 ^cA^

Data are expressed as triplicate mean ± standard deviation. Means with different letters are significantly different at *p* < 0.05 between extracts (uppercase letters) and concentrations (lowercase letters). * Minimum bactericidal concentration of the extract.

**Table 3:** Effective concentrations (EC50) in reducing 50% of 1-diphenyl-2-picrylhydrazyl (DPPH) and 2′-azino-bis-3-ethylbenzthiazoline-6-sulfonic acid (ABTS^•+^) radicals and total phenolic compounds of green coffee bean, coffee silverskin, and PVA extracts.

Extracts	EC_50_ (mg/mL)		Total Phenolics (mg of GAE/g of Extract)
	DPPH	ABTS^•+^	
Green coffee bean	*	5.9 ± 0.1 ^b^	22.3 ± 1.1 ^c^
Coffee silverskin	3.6 ± 0.2 ^b^	6.8 ± 0.1 ^c^	26.9 ± 0.2 ^b^
PVA	0.6 ± 0.03 ^a^	3.2 ± 0.1 ^a^	89.9 ± 2.8 ^a^

Data are expressed as means of triplicates ± standard deviation. Means with different letters are significantly different at *p* < 0.05 by Scott–Knott test. GAE—Gallic acid equivalent. * Value not determined.

**Table 4:** Microcapsule size, encapsulation efficiency, and viability of encapsulated *K. lactis* B10.

Extracts	Microcapsule Diameter (μm)	Encapsulation Efficiency (%)	Viability of Encapsulated *K. lactis* B10 (log CFU/mL)	
			T0	T1
Green coffee bean	1529.49	98.05	8.93	8.76
Coffee silverskin	1581.12	96.51	8.87	8.75
PVA	1451.46	96.32	8.85	8.59

T0—after encapsulation; T1—after passage through simulated gastrointestinal tract.

## Data Availability

The original contributions presented in the study are included in the article, further inquiries can be directed to the corresponding author.
